# Mapping Global Prevalence of *Acinetobacter baumannii* and Recent Vaccine Development to Tackle It

**DOI:** 10.3390/vaccines9060570

**Published:** 2021-06-01

**Authors:** Chaoying Ma, Siobhán McClean

**Affiliations:** School of Biomolecular and Biomedical Sciences, University College Dublin, Belfield, D04 V1W8 Dublin 4, Ireland; chaoying.ma@ucdconnect.ie

**Keywords:** *Acinetobacter baumannii*, antimicrobial resistance, ESKAPE, virulence factors, vaccines, immune response

## Abstract

*Acinetobacter baumannii* is a leading cause of nosocomial infections that severely threaten public health. The formidable adaptability and resistance of this opportunistic pathogen have hampered the development of antimicrobial therapies which consequently leads to very limited treatment options. We mapped the global prevalence of multidrug-resistant *A. baumannii* and showed that carbapenem-resistant *A. baumannii* is widespread throughout Asia and the Americas. Moreover, when antimicrobial resistance rates of *Acinetobacter* spp. exceed a threshold level, the proportion of *A. baumannii* isolates from clinical samples surges. Therefore, vaccines represent a realistic alternative strategy to tackle this pathogen. Research into anti-*A. baumannii* vaccines have enhanced in the past decade and multiple antigens have been investigated preclinically with varying results. This review summarises the current knowledge of virulence factors relating to *A. baumannii*–host interactions and its implication in vaccine design, with a view to understanding the current state of *A. baumannii* vaccine development and the direction of future efforts.

## 1. Introduction

*Acinetobacter baumannii* is an opportunistic gram-negative coccobacillus that demonstrates exquisite survival under various environmental conditions and intrinsic resistance to routinely prescribed antibiotics [1]. Multidrug-resistant (MDR) *A. baumannii* strains are now prevalent worldwide, with the highest percentage of carbapenem resistance of over 90% being reported in the Mediterranean region, imposing serious burdens on healthcare systems. The vast majority of *A. baumannii* isolates arise in medical institutions and are closely associated with nosocomial infections, particularly in patients receiving intensive care and in immunocompromised individuals. Ventilator-associated pneumonia (VAP), central line-associated bloodstream infections (BSI), urinal tract infections (UTI), and meningitis are the most common clinical manifestations [2]. Unfortunately, the lack of efficacious treatments has led to high crude mortality ranging from 40% to 80% for infections occurring in sterile sites [3,4,5,6]. In 2017, the World Health Organization (WHO) listed carbapenem-resistant *A. baumannii* as a critical priority, for which, new antibiotics are urgently needed [7]. In addition, once nosocomial outbreaks of *A. baumannii* occur, it is difficult to thoroughly eradicate from the environment due to its remarkable resistance to disinfectants and its capacity to rapidly develop tolerance to these antibacterial agents, contributing to prolonged colonisation and transmission [8]. Therefore, vaccination of susceptible individuals is likely to be a more effective intervention for the prevention of *A. baumannii* infections. Compared with other nosocomial pathogens such as *Pseudomonas aeruginosa*, *Staphylococcus aureus,* and *Clostridium difficile*, the search for vaccines against *A. baumannii* is in its infancy [9]. Over the last decade, increased research efforts have concentrated on identifying potential virulence factors, clarifying host–pathogen interactions, and developing vaccines against this exquisitely resilient pathogen. In this review, we describe these advances and also provide a comprehensive appraisal of the relevant microbiology, epidemiology, and antibiotic resistance of *A. baumannii*.

**Figure 1:**
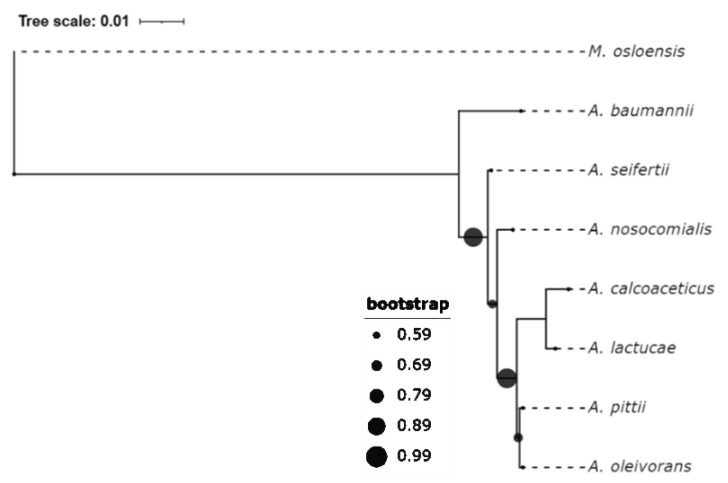
Phylogenetic analysis of the Acb complex. The phylogeny tree was inferred with MEGA X on a Clustal W alignment of the 16S rRNA in the selected type strains. The maximum likelihood-kimura 2-parameter (ML-K2) model was used. *Moraxella osloensis* was chosen as an outgroup. Strain names and their corresponding GenBank accession numbers are listed in Table S1, Supplementary Materials. *A. oleivorans* has also been catalogued into the Acb group in some studies [21,22,23], though its name has not yet been validated.

The genus *Acinetobacter* currently comprises more than 60 validly named species [10]. It has been classified in the family *Moraxellaceae*, within the order *Pseudomonadales*, and the class Gammaproteobacteria since 1991 [11]. The whole genome of *A. baumannii* (strain ATCC 17978) was first sequenced in 2007 by Smith et al. [12]. However, its unambiguous identification in routine diagnostic laboratories has long been hindered as *A. baumannii* is phenotypically similar to, and phylogenetically close to, several other *Acinetobacter* species (Figure 1). The *A. calcoaceticus*–*A. baumannii* (Acb) complex consists of six valid species: *A. baumannii*, *A. calcoaceticus* (formerly *Acinetobacter* genomic species 1), *A. nosocomialis* (formerly *Acinetobacter* genomic species 13TU) [13], *A. pittii* (formerly *Acinetobacter* genomic species 3) [13], *A. seifertii* (formerly *Acinetobacter* genomic species ‘close to 13TU’) [14], and the most recently added *A. lactucae* (formerly *Acinetobacter* NB14, synonymous with *A. dijkshoorniae*) [10,15,16,17]. While *A. calcoaceticus* is non-pathogenic, the other five members are all clinically relevant and are frequently identified in a broad sense as *A. baumannii*, or *A. baumannii* group, in clinical microbial identification. The dominant species among the clinically isolated *A. baumannii* group vary with region [18,19], but from a clinical perspective, *A. baumannii* is the most virulent [5,20].

## 2. *A. baumannii* Is Ubiquitous

*Acinetobacter* spp. are ubiquitous organisms and the first isolate, described as *Micrococcus calcoaceticus* back in 1911, was from soil [24]. Their widely distributed natural reservoirs occupy nearly all environmental niches including waterbodies, soil, mines, crude oil, sewage, sludge, solid surfaces, raw meat, cheese, milk, unprocessed vegetables, human skin, wild animals, livestock, fish, shrimps, plants, and nectar [25]. The natural habitats of *A. baumannii*, however, are still poorly defined as it is almost exclusively isolated from hospital environments and communities involving close contact [2]. *A. baumannii* was rarely detected on human skin (0.5%, *n* = 186), indicating that this is not the natural habitat of *A. baumannii*, despite *Acinetobacter* spp. being associated with human skin flora [26]. The presence of *A. baumannii* in the human body louse has been reported in many countries worldwide [27,28,29]. For example, the highly antibiotic-susceptible *A. baumannii* strain SDF which is responsible for community-acquired infections was first isolated from the interior of body lice collected on homeless people in France [30].

Identification of traces of *A. baumannii* in environmental soil and waterbody samples has frequently been reported [31,32,33,34,35,36,37]. An early study reported that more than a third of the soil samples from Hong Kong contained *Acinetobacter* spp., with *A. baumannii* representing 14.7% (*n* = 34) [38]. More recently, Hrenovic et al. isolated an antibiotic-resistant *A. baumannii* strain from acid paleosol in Croatia [39]. Most water-recovered *A. baumannii* strains were from sewage or urban streams, and hence it is challenging to define whether they are native residents or contaminants from other *A. baumannii* positive fomites [34,35,36,37]. *A. baumannii* colonisation has also been observed in livestock animals, mostly from cattle and poultry [37,40,41,42,43], with various reports in horses, pigs, donkeys, mules, goats, rabbits, dogs, cats, as well as the food products made from them [37,44], but the frequency varies depending on the geographic location of the farms. Wilharm et al. investigated the relatedness of *A. baumannii* isolates from livestock avian in Germany to the lineages spread in hospitals worldwide and found there were several strains closely related to human clinical isolates indicating that livestock avian may serve as a powerful vector for the spread of *A. baumannii* [43]. Considering the complex human–environment–livestock interactions and the elusive transmission chains, these reported cases may be relevant to ‘One Health’ infection control approaches. To date, localised studies have shown considerable variability in the prevalence among birds. For example, an isolation rate of 25% of white stork nestlings in Poland was documented, ranging from 4% to 48% [43]. In contrast, a recent regional study demonstrated *A. baumannii* barely existed in the choana and rectums of wild songbirds and gulls captured in Germany and Poland (0.19%, *n* = 1051) [45]. More culture-based screenings of avian populations worldwide, including their living environments are necessary. Genomic approaches may facilitate the linkage between environmental strains and clinical isolates in time excluding misleading strains, that are hospital-derived but contaminating other places.

## 3. Antimicrobial Resistance

**Figure 2:**
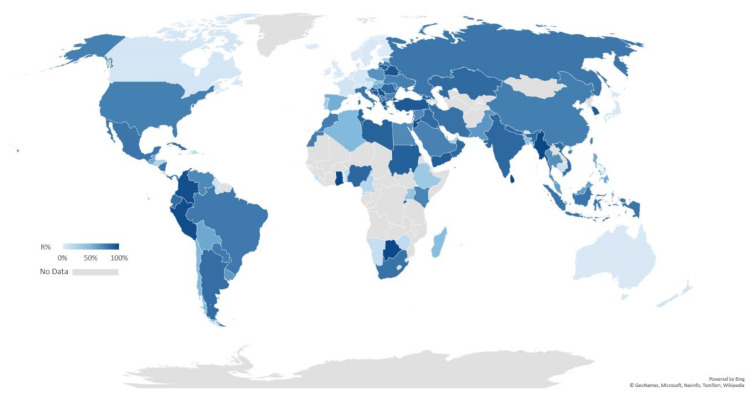
Global percentage of resistance to carbapenems (R%) of *A. baumannii*. The shade of blue represents the level of carbapenem resistance worldwide during the last decade (2009–2018). No suitable surveillance or research data is available for areas coloured in grey. A full list of all the statistics and sources is provided in Table S2, Supplementary Materials.

The antimicrobial resistance of *A. baumannii* is of major concern. It is classified as an ESKAPE pathogen, an acronym referring to *Enterococcus faecium*, *S. aureus*, *Klebsiella pneumoniae*, *A. baumannii*, *P. aeruginosa*, and *Enterobacter* spp., all of which are recognised nosocomial bacteria with high potential to exhibit MDR and virulence [46]. Clinical isolates *A. baumannii* strains are frequently reported to demonstrate resistance to the most routinely prescribed antibiotics in varying degrees, including carbapenems such as imipenem and meropenem. Carbapenem is one of the β-lactam antibiotics with the broadest spectrum and highest efficiency. Resistance to carbapenem is considered to be a marker for extensively resistant bacteria because it involves a broad range of co-resistance to unrelated antibiotic classes [7]. We have collated an up-to-date profile on the global incidence of carbapenem-resistant *A. baumannii* strains documented over the past decade (Figure 2). The parameter R% was defined as the percentage of carbapenem-resistant isolates among all clinically isolated *A. baumannii* strains. Data for each country or region were collected from their latest antimicrobial resistance (AMR) surveillance annual reports, and in the case of those without applicable surveillance data, published figures from the most recent research or reviews were used. It is apparent that carbapenem-resistant *A. baumannii* is now problematic across Asia and the Americas, except in Japan and Canada. Moreover, Oceania, Western Europe, the Nordic region, and part of central Europe have the lowest R% (<10%), however, in areas surrounding the Mediterranean, including southern Europe, the Middle East, North Africa, up to 90% of *A. baumannii* clinical isolates are resistant to carbapenems. Details for central Africa and island countries are limited.

In the treatment of *A. baumannii* infections, MDR often compels the adoption of last-resort antibiotics such as colistin when essentially no other options are available. However, the increasing quantity of recent cases reporting the emergence of pan-drug-resistant (PDR) strains tolerant to last-resort therapies has dimmed their potential [47,48,49,50,51,52].

## 4. Clinical Importance

### 4.1. Susceptible Populations

*A. baumannii* has been long been recognised as a major opportunistic pathogen causing nosocomial infections, or healthcare-associated infections, and manifests as a broad range of infections, including respiratory infections, BSIs, UTIs, and meningitis. [2]. Patients admitted to intensive care units (ICU), patients with indwelling medical devices, or with immunocompromised medical conditions are particularly predisposed to its infections. Falagas et al. demonstrated that the length of ICU stay was significantly increased in patients with *A. baumannii* infections [53]. Previous studies demonstrated that the crude mortality rate of *A. baumannii* infection was over 50% [3], although the attributable mortality varies with infection type. For example, in a multicentre study conducted in eight US metropolitan areas from 2012–2015, the overall recorded death rate of carbapenem-resistant *A. baumannii* infections was 17.9%, of which infections occurring at normally sterile sites had much higher mortality of 41.3% compared with UTIs (8.3%) [3]. *A. baumannii* is one of the leading causes of VAP worldwide, especially in Asia, Latin America, and the Middle East [54]. The ICU mortality of MDR *A. baumannii* caused VAP has been reported to be as high as 84.3% [4]. Crude mortality in patients with *A. baumannii* bacteraemia ranges from 37% to 52% in the U.S. [5]. Although the incidence of *A. baumannii* meningitis is comparably low, it is an increasing threat in post-neurosurgical patients, with a mortality rate approaching 70% [6]. *A. baumannii* skin and soft tissue infections in trauma (wound, burn) victims following natural disasters or wars have also been recorded, such as those during the Iraq conflicts, the Syrian war, the Wenchuan earthquake, the Marmara earthquake, and the Indian Ocean tsunami [47,55,56,57,58]. According to a single site study, after the Wenchuan earthquake, 14% of wound infections in the hospitalised survivors were caused by *A. baumannii* [58]. Noticeably, during the Coronavirus disease 2019 (COVID-19) pandemic, hospital-acquired co- or secondary infections in COVID-19 in-patients caused by *A. baumannii* were reported worldwide [59,60,61]. An acute care hospital in New Jersey reported 70% (14/20) of patients with hospital-acquired *A. baumannii* infections developed VAP and 85% (17/20) of them had co-infections with SARS-CoV-2 [61]. In a serious outbreak in Texas, COVID-19 patients with secondary *A. baumannii* infections experienced a two-fold higher mortality rate relative to COVID-19 patients without *A. baumannii* infections. These reports suggested that *A. baumannii* co-infection may exacerbate the development and prognosis of COVID-19 and hinder the clinical diagnosis and treatment [62].

Community-acquired *A. baumannii* infections are rarely reported but comparably more often in some tropical or sub-tropical areas in the Asia–Pacific such as northern Australia and Taiwan [63,64,65,66], indicating the potential climate drivers of its prevalence [67]. Unlike healthcare-associated infections, *A. baumannii* strains involved in community-acquired infection are more susceptible to antibiotics. Farrugia et al. sequenced the whole genome of a community strain D1279779 obtained from Darwin, Australia, and found that the antibiotic resistance island AbaR, associated with nosocomial strains, was absent in that strain [68]. Despite their low occurrence, community-acquired *A. baumannii* infections are characterised by an acute fulminant course involving septic shock, respiratory failure, severe sepsis, pneumonia, and the mortality can be quite high, i.e., over 60% [67,68].

### 4.2. Prevalence

**Figure 3:**
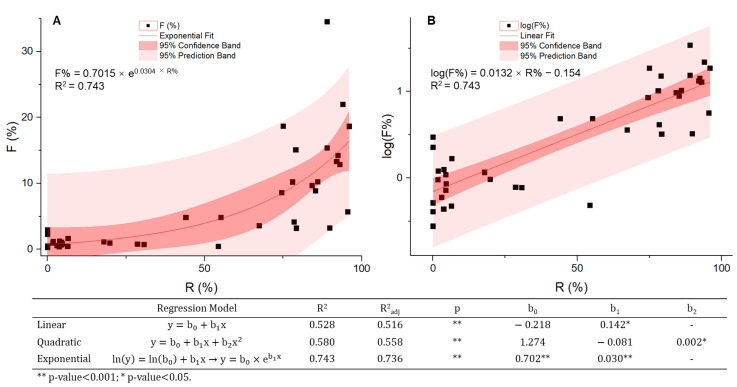
Correlations between R% and F%. Correlation analysis and curve estimation were performed with SPSS and presented on linear (**A**) and logarithmic (**B**) scales. Both data sets follow non-normal distributions, and the correlation coefficient was calculated using Spearman’s rank-order method. R% and F% are in strong positive correlation with the best fit in the exponential model.

Since the 1990s, outbreaks of *A. baumannii* in clinical sites have often been reported [69], particularly in regions with high levels of carbapenem-resistant rates. In Europe, the clinical isolation rate ranges from less than 1% to over 30% [70,71], and clinical outbreaks are most intensively reported in eastern and southeast Europe. We defined F% as the proportion of *Acinetobacter* spp. isolates identified among all the clinical samples of monitored AMR species (*Escherichia coli*, *K. pneumoniae*, *P. aeruginosa*, *Acinetobacter* spp., *Streptococcus pneumoniae*, *S. aureus,* and *Enterobacter* spp.). The parameter F% was compared to R% by region (Figure 2) using the 2018 data from two international surveillance networks, the European Antimicrobial Resistance Surveillance Network (EARS-Net) initiated by the European Centre for Disease Prevention and Control (ECDC) and the Central Asian and European Surveillance of Antimicrobial Resistance Network (CAESAR) from WHO, which together covered the entire European data where both the world’s lowest (<1%) and the highest resistant rates (>95%) can be found (Table S3, Supplementary Materials). There is a strong positive correlation between the two variables with a Spearman’s rank correlation coefficient (ρ) of 0.808 (*p*-value of 0.000). Curve estimation (Figure 3) shows the data points fit best in the exponential regression model described by the equation of F% = 0.7015 × e^0.0304×R%^ (adjusted R^2^ = 0.736). This exponential model suggests that when antibiotic resistance rates are low in a region, the clinical isolation rates of *Acinetobacter* spp. generally remain relatively stable, but once the R% exceeds a certain level, the proportion of *A. baumannii* isolates from clinical samples surges, a stark warning that there might be considerably more *A. baumannii* associated infections and a higher risk of nosocomial outbreaks in the future. In this case, with an R% of less than 10%, the detecting frequency F% can be as low as <1%; whereas F% increases by ten-fold if the R% is more than 90%. However, due to the small sample size (*n* = 41) and high variance, the relationships between the prevalence of *A. baumannii* outbreaks and its antibiotic resistance should be further validated with more relevant data. No evidence of gender or age difference in incidence has been reported so far.

Regional differences in the overall proportion of *A. baumannii* among all clinical isolated, aerobic, and facultative gram-negative pathogens have been reported, ranging from 0.7% in North America to 4.6% in the Middle East [72]. The Middle East is one of the areas hardest hit by *A. baumannii*. Historically, *A. baumannii* was also known as ‘Iraqibacter’ because it caused outbreaks of MDR strains in the US military hospitals based in Iraq, Kuwait, and Afghanistan during the Iraq War [55,73]. These strains then spread outside the army to the civilian health care system, most probably via the transfer of colonised casualties [1,55], and have continued to be a persistent issue in these areas. Unfortunately, although serious infections and mortality caused by MDR *A. baumannii* have been reported [47,51,74], regional epidemiology studies are quite limited. A recent MLST-based study showed that sequence types ST2, ST195, and ST208 had been repeatedly reported from many Middle Eastern countries [75]. Moreover, US service members continue to be colonised with ‘Iraqibacteria’ and, although controversial, they may serve as reservoirs participating in the community transmission [76,77]. For example, a record review performed on the prevalence of MDR pathogens in a burn centre dedicated to military personnel reported that *A. baumannii* was the most prevalent organism recovered (22%) during the study period of 2003–2008 [78]. Subsequently, from 2009 to 2013, an equivalent level was reported in a study of nationwide data [79]. In contrast, the proportion was approximately 10% before 2000 [80], suggesting that *A. baumannii* has spread from the US military system to the civilian settings.

## 5. Virulence Factors

Severe tissue damage with infiltration of large numbers of inflammatory cells is frequently observed at *A. baumannii* infectious sites. High levels of interleukin 2 (IL-2), IL-4, IL-17, tumour necrosis factor α (TNF-α) and interferon γ (IFN-γ), as well as a lower CD4+/CD8+ ratio, have been reported [81]. The virulence of *A. baumannii* is strain-dependent and involves complicated mechanisms of pathogenicity which are only beginning to be elucidated. Many efficacious vaccines against other pathogens have been developed based on virulence factors, consequently, *A. baumannii* virulence factors are worth reviewing.

### 5.1. Adhesins and Invasins

Studies have demonstrated that the respiratory epithelial cells are more easily invaded by *A. baumannii* than non-respiratory epithelial cells [82], which likely contributes to the high occurrence and mortality rate of *A. baumannii* pneumonia. Outer membrane proteins (OMP), including OmpA (also known as Omp38), are the most well-studied *A. baumannii* adhesins [82,83,84,85] (Table 1). OmpA is a conserved, abundant porin in *A. baumannii* with a molecular weight of 38 kDa and plays an essential role in adhesion and invasion. Competitive binding studies with recombinant *A. baumannii* OmpA attenuated both cell binding and invasion [82]. *A. baumannii* attaches with moderate affinity to epithelial cells, secreting OmpA into the cells, which leads to bacterial uptake via actin rearrangement and membrane reorganisation of host cells, known as a zipper-like mechanism [82]. The internalised bacteria are located in membrane-bound vacuoles [82] and the internalised OmpA is translocated to the nucleus and mitochondria, causing the release of a series of proapoptotic molecules, which promotes cell apoptosis [83,84]. Consequently, OmpA also acts as a toxin contributing to pathogenesis. Notably, we ran BLASTp against the Swiss-Prot database and found that the *A. baumannii* OmpA (UniProt ID: Q6RYW5) has no significant homology with *E. coli* (K12) OmpA (96% query cover, 23% identities) but shares 46% and 41% identities with probable lipoprotein YiaD and YfiB, respectively. Considering the query covers are relatively low (31% for both), the results should be interpreted cautiously. Similarly, Omp33–36 (also known as Omp34) is another outer membrane virulence factor involved in host–cell attachment. It is also cytotoxic, inducing apoptosis by activation of caspases and modulation of autophagy, with the consequent accumulation of sequestosome 1 and autophagosome LC3B-II [86]. An isogenic *A. baumannii* mutant deficient in the *omp33* gene resulted in a significant reduction in adherence, invasion, and cytotoxicity of human lung epithelial cells, compared to the wild type [86,87]. Other OMPs functioning as *A. baumannii* adhesins include the biofilm-associated protein (Bap), which increases the membrane hydrophobicity [88]; the FhaB/FhaC and CdiA/CdiB type Vb secretion system [89]; and the *Acinetobacter* trimeric autotransporter (Ata). Ata mediates adherence through binding to type IV collagen in the extracellular matrix (including basal lamina) of host cells [90,91,92]. It also contributes to cytotoxicity by inducing the secretion of pro-inflammatory cytokines, such as IL-6 and IL-8, and leading to cellular apoptosis in a caspase-dependent manner [92].

Recently, non-OMP adhesins and invasins have been identified. Both the response regulator, BfmR, and the sensor kinase, BfmS, in the BfmRS two-component system have been reported to play a role in cell adherence [93,94]. Several pili have been found to be relevant with the attachment to both biotic and abiotic surfaces, indicating that the motility may correlate with the adherence ability. Wood et al. showed that *A. baumannii* is capable of producing different adhesins and pilus assembly systems in response to changes in environmental conditions, such as the BlsA mediated, photoregulated type I pilus assembly system PrpABCD [95]. Eijkelkamp et al. found that type IV pili, associated with twitching motility, may also mediate adhesion [96]. Interestingly, they later found the expression of a homologue of the histone-like nucleoid structuring (H-NS) protein, a known global transcriptional repressor, was disrupted in an *A. baumannii* strain with enhanced adherence and motility [97]. Thioredoxin-A protein (Trx-A) is a mediator of the type IV pilus system and hence, is also involved in host–cell adhesion [98]. In contrast, the chaperone-usher (Csu) type I pili does not play a role in adherence [99]. Phospholipase C (PLC) and phospholipase D (PLD) both play significant roles in invasion and their inactivation led to impaired epithelial cell invasion and a reduction of the extrapulmonary tissue bacterial burden [100,101,102]. OMPs containing phosphorylcholine (ChoP) are also involved in bacterial adherence and invasion [103]. *A. baumannii* bound via ChoP to the platelet-activating factor receptor (PAFR) on human lung cells, resulting in activation of signalling involving G protein, clathrin, β-arrestins, proteins involved in the direction of the vacuolar movement, and intracellular calcium, which eventually led to invasion [103]. Host cell receptors are poorly understood. Johansson et al. recently investigated the potential glycoconjugate receptors by binding *A. baumannii* to various glycosphingolipids on thin-layer chromatograms [104]. Collectively, three predominant glycosphingolipid receptors were isolated and identified, neolactotetraosylceramide, lactotetraosylceramide, and lactotriaosylceramide, indicating that N-acetylglucosamine (GlcNAc) was the basic recognition pattern [104].

### 5.2. Cytotoxins

The envelope of *A. baumannii*, particularly lipooligosaccharides (LOS), contributes to its cytotoxicity towards host cells. LOS is the major component on the outer leaflet of the outer membrane [105] and contributes to both immunogenicity and cytotoxicity of *A. baumannii*. Erridge et al. showed that *A. baumannii* LOS stimulates TNFs and IL-8 secretion from macrophages [106]. Several studies have demonstrated that the absence or disruption of any of the first three enzymes in the lipid A biosynthetic pathway, acyltransferase (LpxA), deacetylase (LpxC), and N-acyltransferase (LpxD), results in less-lethal infections and reduced serum survival [107,108,109,110], highlighting the immunogenicity of *A. baumannii* LOS.

As previously mentioned, several envelope proteins including OmpA, Omp33–36, and Ata, also contribute to cytotoxicity. Bacterial γ-glutamyl transferase enzyme (GGT), secreted via the type II secretion system, was also reported to cause cell damage directly by caspase activation and apoptosis, depletion of ATP and subsequent necrosis, and cell-cycle arrest. A high level of serum GGT was associated with oxidative stress and the exacerbation of chronic obstructive pulmonary disease (COPD) [111,112]. Very recently, Elhossieny et al. characterised *A. baumannii* GGT and showed *A. baumannii* strains with higher extracellular GGT activity resulted in more severe tissue damage by inducing increased inflammation and oxidation activities with elevated phenoloxidase, lysozyme, lactate dehydrogenase, and lipid peroxidation observed [113].

There are additional virulence factors whose roles and molecular interactions are not fully understood, but their absence has resulted in impaired virulence or lethality. For example, Smith et al. reported a type IV secretion system may contribute to the pathogenesis of *A. baumannii* based on its identification within pathogenicity islands (PAI) after whole-genome sequencing, but its specific roles have not been studied to date [12]. Further, it has been suggested that *A. baumannii* may utilise a type VI secretion system to kill competing bacteria [114].

### 5.3. Persistence

In addition to adhesion, invasion, and cytotoxicity contributing to *A. baumannii* pathogenesis, serum persistence also affects the infection process indirectly. Capsular exopolysaccharide (CPS) is an envelope component mediating the persistence of many invasive bacteria, enabling organisms to survive better under unfavourable conditions, e.g., desiccation. It also effectively protects pathogens from phagocytosis and humoral immune attack and inhibits the activation of the alternative complement pathway [115]. Many clinical isolates of *A. baumannii* express the K locus gene cluster, responsible for the biosynthesis and export of CPS [105,116]. However, CPS is not usually, in itself, a pathogenicity determinant as many organisms possessing polysaccharide capsules do not cause human diseases. Russo et al. identified two capsule associated genes, *ptk* and *epsA*, that were predicted to encode a putative protein tyrosine kinase (PTK) and a putative polysaccharide export OMP (EpsA), respectively, and the *ptk* and *epsA* mutants showed significantly decreased survival in soft tissue infection sites [117]. Similarly, *pglC* and *pglL* mutants showed impaired survival in mouse septicaemia models [118,119], suggesting that CPS may act more as a protectin, maintaining serum resistance during the infection process, rather than cytotoxin.

LOS may be important for *A. baumannii* serum resistance, conferring a competitive advantage in vivo, as mutants lacking LpsB, a highly conserved glycosyltransferase involved in LOS synthesis, showed reduced resistance to human serum [107]. Metal acquisition systems, such as iron (BasD, BauA, NfuA), zinc (ZnuABC, ZigA), and manganese (MumC, MumT) acquisition systems also contribute to in vivo survival [101,120,121,122,123]. Koenigs et al. identified a novel plasminogen binding protein, CipA, the absence of which resulted in the efficient killing of *A. baumannii* by human serum and impaired its ability to penetrate the endothelial monolayers [124]. Universal stress protein A (UspA) has also been documented to play a role in protecting *A. baumannii* from H_2_O_2_, low pH, and 2,4-DNP, and the *uspA* mutant formed fewer colonies in sepsis or pneumonia mouse models [125,126,127]. The capacity of *A. baumannii* to confer resistance to and degrade H_2_O_2_ is also largely dependent on the catalases KatE and KatG, which may attenuate the production of reactive oxygen species (ROS) by phagocytic cells of the innate immune system [128]. Finally, OmpA, phospholipase, BfmR/S system, Tuf, and RecA, have also been shown to contribute to persistence [129].

**Table 1:** Identified *A. baumannii* Virulence Factors and Associated Functions.

Virulence Factors	Functions Involved	Adhesion	Invasion	Cytotoxicity	Ref
Ata	type Vc secretion system	+	+	+	[90,91]
Bap	type I secretion system	+			[88]
BasD, BauA	iron acquisition system		+	+	[130]
BfmR/S	two-component regulatory system	+		+	[93,94]
BLP-1, BLP-2	Bap like protein	+			[131]
Capsular polysaccharides	outer membrane component	+			[117]
CdiA/CdiB	type Vb secretion system	+			[132]
ChoP	phosphorylcholine	+	+		[103]
CipA	plasminogen binding protein		+		[124]
FhaB/FhaC	type Vb secretion system	+			[89]
GGT	type II secretion system	+		+	[113]
LOS	outer membrane component			+	[106,107]
LpsB	LOS production			+	[107]
LpxA, LpxC, LpxD	LOS production			+	[107,108,109,110]
NfuA	iron acquisition system		+	+	[101,120,122]
OmpA	porin	+	+	+	[82,83]
Omp33-36	porin	+	+	+	[86,87]
paaE	production of toxic epoxide compounds			+	[129,133]
PLC, PLD	phospholipase		+		[100,101,102]
TrxA	type 4 pili production	+			[98]
Type 4 pili	motility apparatus	+			[96]
T6SS	type VI secretion system		+	+	[114,134]

## 6. Anti-*A. baumannii* Vaccine Development

With the extensive spread of MDR *A. baumannii* worldwide and increasingly frequent reports of emerging PDR strains, more effective control implications are urgently needed to combat this imminent threat. Thus, novel antibiotics and anti-*A. baumannii* vaccines are the two main routes being followed, with the latter being especially important in view of the likelihood of the development of resistance to new antibiotics. *A. baumannii* has an intracellular lifestyle, invading host cells via a zipper-like mechanism [82] and therefore *A. baumannii* vaccines should induce acquired cellular immunity with long-term memory [135], while those that can elicit both humoral and cellular immune memory are expected to be optimal. Over a decade ago, McConnell et al. pioneered their development [136,137,138], and currently, several laboratories around the world focus on developing *A. baumannii* vaccines for targeted populations.

### 6.1. Target Populations

As an opportunistic pathogen, *A. baumannii* rarely causes diseases in healthy people, nevertheless, protection is needed for vulnerable individuals, particularly those residing in, or frequently travelling to, areas with a high prevalence or historic outbreaks of *A. baumannii*. These susceptible cohorts are the main target population of *A. baumannii* vaccines and were nominally identified and characterised into three groups (Table 2) based on reported epidemiology studies.

### 6.2. Vaccine Candidates

Scientists have been pursuing an effective vaccine against *A. baumannii* for the past decade and several antigens and strategies have been examined in preclinical studies.

#### 6.2.1. Whole-Cell Vaccines

Given that a considerable proportion of the individuals susceptible to *A. baumannii* are those with compromised health conditions, there are perceived potential safety risks with administering live-attenuated vaccines to vulnerable cohorts. Consequently, to our knowledge, only one attenuated whole-cell *A. baumannii* vaccine candidate has been reported to date which comprises a genetically modified, attenuated MDR clinical strain deficient in the TrxA adhesin (Table 3). Mice immunised via intraperitoneal (i.p.) or subcutaneous (s.c.) routes were protected by 100% and 90% respectively against a ten-fold lethal dose (LD_50_) challenge for a period of four weeks post-challenge. Moreover, immunisation-induced antisera provided fully effective protection in passive immunisation experiments, highlighting that this attenuated live vaccine induced robust humoral responses despite minimal splenic cell-mediated immunity [139].

Several inactivated whole-cell vaccine candidates formulated either with or without adjuvants were also evaluated [137,140,141,142]. The first reported vaccine candidate against *A. baumannii* was a formalin-killed ATCC 19606 whole-cell vaccine administered with aluminium phosphate adjuvant. Mice intramuscularly (i.m.) immunised with this vaccine were effectively protected from a lethal sepsis challenge raised by heterologous clinical strains, including one PDR strain, with seven-day survival rates between 85%–100%. Significant increases in the serum levels of immunoglobulin G1 (IgG1), IgG2a, and IgM were detected, suggesting a mixed Th1/Th2-mediated response was stimulated [137]. Interestingly, while sepsis is the most frequently used challenge model, KuoLee et al. first reported an experimental vaccine for protection against respiratory challenge, to test a candidate administered via mucosal route. This intranasal (i.n.) immunisation with formalin-killed cells of a hypervirulent *A. baumannii* strain LAC-4 successfully elicited both mucosal and humoral immune responses in mice with significant levels of IgA, IgG1, and IgG2a in bronchoalveolar lavage (BAL) fluid being achieved. All immunised mice were completely protected from lethal i.n. or i.p. challenges raised by the LAC-4 strain for up to 10 days [141].

#### 6.2.2. Subunit Vaccines

As novel virulence factors of *A. baumannii* and their molecular interactions continue to be identified in the past decade, their immunogenicity and their potential as subunit vaccines have been more extensively investigated (Table 3). Outer membrane immunogenic proteins functioning as adhesins or invasins are the main targets for vaccine development because they represent the initial stages of infection, namely bacterial attachment to host cell receptors and colonisation, and blocking this may be the most effective strategy to prevent bacterial infections [143].

Probably the most understood and promising subunit candidate for *A. baumannii* vaccines is OmpA because, as previously discussed, it is an all-round player involved in almost every critical step of *A. baumannii* pathogenesis, including adhesion, invasion, cytotoxicity, and also in vivo persistence, and is highly immunogenic in animal models. Elucidation of the native structure of *A. baumannii* OmpA showed that its amino acid sequence has no homology to the human proteome but shows a low variation (≥89% conserved) across clinical isolates [144,145], which indicates that *A. baumannii* OmpA would be less likely to cause mutual interference with other OmpA-containing commensal bacteria, minimising the potential side effects as a vaccine. Several studies have presented positive results demonstrating active immunisation with OmpA-based vaccines could confer prophylactic benefits against *A. baumannii* (Table 3*)*. Intranasally immunised rOmpA conjugated to cholera toxin, showed 15-day survival rates from 40% to 100% following i.p. challenges compared with 100% mortality in two days in the control groups [146]. A study in older (>6 months) diabetic mice demonstrated protection of 50% for up to 28 days following rOmpA vaccination which was equivalent to that in the juvenile group [147] suggesting that OmpA-based vaccine protection may translate across a broad age range and include people with diabetes. The dose of antigen administered may impact the profile of the immune response with low dose (3 μg) rOmpA immunised mice showing a balanced IFN-γ and IL-4 immune responses, while mice that received a higher dose (100 μg) showed a polarised Th2-response as demonstrated by IL-4 secretion [148].

Besides OmpA, several other virulence factors have also been tested as prospective vaccine candidates. Omp33–36 was specifically recognised by IgG, IgM, and IgA from *A. baumannii* colonised patients and showed no cross-reaction with sera from other infections, [149], highlighting its potential as a vaccine antigen. In addition, this highly conserved adhesin is present in over 1600 strains of *A. baumannii* with ≥98% identity [150]. Omp33–36 is considered as a suitable immunogen to protect against *A. baumannii* and its co-administration with OmpA may confer even greater protection; however, data from active immunisation studies are currently insufficient. In silico simulation also predicted peptides constructed by exposed epitopes of Omp33–36 could trigger antibody responses with higher avidity [150]. Alternatively, Bentancor et al. reported the passive administration of anti-Ata rabbit sera protected both immunocompetent and immunocompromised mice from *A. baumannii*-induced pneumonia by engendering anti-adhesive and robust opsonic activities [151], making Ata a potential vaccine antigen. Although Ata has been identified in many clinical isolates, the variable expression of Ata among clinical isolates suggests that it may not provide adequate coverage [17] as a monovalent vaccine. CPS PNAG and K1 were also suggested as targets for protective immunity against *A. baumannii* infections [152,153] but their efficacy, when conjugated with carriers, has not been studied yet. Recombinant Bap completely protected immunised mice from sepsis following a lethal challenge, and when mice were challenged at the extreme dose of 10^6^ × LD_50_ (10^13^ CFU), a five-day survival rate of 60% was obtained [154]. It should be noted that the use of Freund’s adjuvant (FA) as adjuvant undermines the relevance of this response, given its reactogenicity. Two siderophore receptors in *A. baumannii*, BauA, and BfnH, proved only moderately effective against lethal sepsis challenges with survival rates of 40% and 44.5%, respectively [155].

**Table 3:** Preclinical active immunisation studies of *A. baumannii* vaccine candidates.

Platform and Immunogen	Mice Strain	Immunisation Schedule			In Vivo Challenge Study					Immune Responses	Ref.
		Day	Route	Dose	Chall. Strain(s)	Model (Route)	Dose * (CFU) (LD_50_)	Survival (%)	Survival monitored (days post challenge)		
*Live Attenuated Vaccines*											
Ci79 Δ*trxA*	C57BL/6 (4w–6w)	0, 14	i.p. / s.c.	2 × 10^5^ CFU	WT Ci79	day 28 sepsis (i.p.)	5 × 10^6^ (10 × LD_50_)	i.p.—100% s.c.—90%	28 days	robust humoral responses, minimal cellular immunity	[139]
							1 × 10^6^ (2 × LD_50_)	i.p.—100% s.c.—100%			
*Inactivated Vaccines*											
formalin-killed ATCC 19606 with AlPO_4_	C57BL/6 (6w–8w)	0, 21	i.m.	1 × 10^8^ CFU	ATCC 19606 Ab-154 113-16	day 28 sepsis (i.p.)	1.6 × 10^6^ (242 × LD_50_) 3.1 × 10^6^ (6.5 × LD_50_) 4.1 × 10^6^ (74.5 × LD_50_)	100% 100% ~85%	7 days	significant levels of IgG and IgM, Th2- and Th1-mediated responses	[137]
formalin-killed LAC-4	C57BL/6 BALB/c (8w–12w)	0, 14, 21	i.n.	5 × 10^7^ CFU	LAC-4	day 42 sepsis (i.p.) pneumonia (i.n.)	8.4 × 10^5^ (i.p.) 7 × 10^7^ (i.n.)	100%	10 days	mucosal immune responses, dominant Th2 responses	[141]
formalin-killed IB010 (ATCC 19606 Δ*lpxD*) with AlPO_4_	C57BL/6 (6w–8w)	0, 14	i.m.	1 × 10^9^ CFU	ATCC 19606 Ab-154	day 21 sepsis (i.p.)	2.3 × 10^6^ (341 × LD_50_) 1.1 × 10^6^ (2.2 × LD_50_)	100% 100%	7 days	robust IgG1 and IgG2c responses	[142]
*Subunit Vaccines*											
rOmpA (ATCC 19606) conjugated with cholera toxin	BALB/c (6w–8w)	0, 21	i.n.	10 μg	strain A strain B strain C strain D strain E	day 28 sepsis (i.p.)	5 × 10^8^ (2.5 × LD_50_) 2 × 10^8^ (2 × LD_50_) 5 × 10^7^ (2 × LD_50_) 3 × 10^8^ (3 × LD_50_) 3 × 10^7^ (3 × LD_50_)	~50% ~40% ~55% 100% ~70%	15 days	significant levels of IgG and IgA	[146]
rOmpA (ATCC 17978) with Al(OH)_3_	diabetic BALB/c (>6 m and 6w–10w)	0, 21	s.c.	3 μg	HUMC 1	day 35 sepsis (i.v.)	2 × 10^7^	~50%	28 days	robust humoral responses	[147]
rBap (Kh0060) with FA	BALB/c (4w–6w)	0, 14,28	not stated	10 μg	Kh0060	day 35 sepsis (i.p.)	1 × 10^9^ (100 × LD_50_) 1 × 10^11^ (10^4^ × LD_50_) 1 × 10^13^ (10^6^ × LD_50_)	100% 80% 60%	5 days	robust humoral responses	[154]
rBauA or rBfnH (ATCC 19606) with FA	BALB/c (6w–8w)	0, 14,28	s.c.	20 μg	ATCC 19606	day 49 sepsis (i.p.)	1.5 × 10^7^ (2 × LD_50_)	~40%	7 days	not specified	[155]
rBamA (ATCC 19606) with Al(OH)_3_	BALB/c (6w–8w)	0, 14,28	i.p.	20 μg	P-562	day 45 pneumonia (i.n.)	1 × 10^9^	~80%	7 days	non-neutralising, opsonic IgG	[156]
rBamA (ATCC 19606] with FA	BALB/c (6w–8w]	0, 14, 28, 42	s.c.	20 μg	ATCC 19606	day unknown, sepsis (i.p.)	2 × 10^6^ (4 × LD_50_) 7 × 10^6^ (14 × LD_50_)	100% 25%	4 days	not specified	[157]
rTrx-OmpW (ATCC 17978) with alum	ICR (6w–8w)	0, 14,28	s.c.	50 μg	ATCC 17978	day 49 sepsis (i.p.)	1 × 10^6^	100%	7 days	opsonic IgG, complements	[158]
rTrx-Omp22 (ATCC 17978)	ICR (6w–8w)	0, 14, 28	s.c.	50 μg 20 μg 10 μg 5 μg	Ab1	day 49 sepsis (i.p.)	1 × 10^6^	100% 33% 33% 0	7 days	opsonic IgG, complements	[159]
rOmp22 (ATCC 19606) with FA	BALB/c (6w–8w)	0, 14,21	s.c.	20 μg	ATCC 19606	day 42 sepsis (i.p.)	2 × 10^8^	37.5%	8 days	not specified	[160]
rOmpK (ATCC 19606) with FA	BALB/c (6w–8w)	0, 14,21	s.c.	20 μg	ATCC 19606	day 42 sepsis (i.p.)	2 × 10^8^	25%	8 days	not specified	[160]
rOmp22-OmpK (ATCC 19606) with FA	BALB/c (6w–8w)	0, 14,21	s.c.	20 μg	ATCC 19606	day 42 sepsis (i.p.)	2 × 10^8^	67%	8 days	not specified	[160]
rOmp22-OmpK (ATCC 19606) with MF59 adjuvant	BALB/c (6w–8w)	0, 14,21	i.t.	30 μg	ATCC 19606	day 42 respiratory (i.t.)	1 × 10^8^	83.3%	10 days	significant levels of IgG and IgA	[161]
*Multi-Component Vaccines*											
OMC (ATCC 19606) with AlPO_4_	C57BL/6 (6w–8w)	0, 21	i.m.	25 μg	ATCC 19606 Ab-154 113-16	day 35 sepsis (i.p.)	1 × 10^6^ (151.3 × LD_50_) 1 × 10^7^ (20.9 × LD _50_) 1.5 × 10^6^ (27.3 × LD_50_)	100% 80% 100%	7 days	robust IgG and IgM responses	[136]
OMV (ATCC 19606) with AlPO_4_	C57BL/6 (6w–8w)	0, 14	i.m.	10 μg	ATCC 19606 Ab-154 113-16	day 35 sepsis (i.p.)	4.5 × 10^5^ (71.3 × LD_50_) 1.7 × 10^6^ (4.3 × LD_50_) 2 × 10^6^ (39.9 × LD_50_)	100% ~90% 100%	7 days	robust IgG1, IgG2c and IgM responses	[138]
ClyA-Omp22 (ATCC 17978) with E. coli derived OMV	ICR (6w–8w)	0, 14	s.c.	5 μg 10 μg 20 μg 50 μg	Ab1	day 35 sepsis (i.p.)	1 × 10^6^	30% 40% 63.6% 100%	7 days	opsonic IgG	[162]
*Nucleic Acid Vaccines*											
pVAX1-*ompA* (LAC-4) with CpG-ODN adjuvant	C57BL/6 (6w–8w)	0, 14, 21	i.m.	100 μg	LAC-4 SJZ04 SJZ18 SJZ28	day 28 respiratory (i.t.)	1.2 × 10^7^ (2 × LD_50_) 1 × 10^8^ 1 × 10^8^ 1 × 10^8^	~50% ~40% ~60% ~50%	7 days	robust humoral responses, mixed Th1/Th2/Th17 cellular responses	[163]
pBudCE4.1-*ompA*	BALB/c (6w–8w)	0, 7, 14, 28	i.m.	25 μg	not stated	day 35 respiratory (i.n.)	1 × 10^8^	60%	15 days	significant levels of IgG and IgM	[164]

Abbreviations: i.m., intramuscular; i.n., intranasal; i.t., intratracheal; i.p., intraperitoneal; s.c., subcutaneous. * Dose includes LD50, when reported.

Additionally, some OMPs which are less relevant to *A. baumannii* pathogenesis and overlooked as virulence factors may also have a potential impact as vaccines. These immunogens are likely to be safer even when a high dose is given [159] and have been shown to be efficacious antigens in other infections [165]. Purified renatured OMP assembly factor BamA protected mice against a lethal pneumonia challenge with a seven-day survival rate of 80% and 60%, respectively following active and passive immunisation. The opsonophagocytic killing assay showed the immunisation elicited a high titre of non-neutralising, opsonising antibodies [156]. The carbapenem resistance OMP (CarO) was also predicted to contain structurally important linear B cell epitopes based on bioinformatic design [166]. OmpW is an *A. baumannii* porin involved in iron uptake and colistin binding which is conserved across 804 strains with more than 91% identity in protein sequence [167]. We have previously reported that the *Burkholderia pseudomallei* OmpW homolog afforded extended protection (81 days) against a lethal melioidosis challenge [165]. Immunisation with a recombinant Trx–OmpW fusion protein fully protected mice from sepsis challenge for up to seven days. The antisera demonstrated significant opsonophagocytic activities against homologous strains and clonally distinct clinical isolates in vitro [158]. Later, the same research group identified a novel OMP with a molecular weight of 22 kDa, namely Omp22, highly conserved (95% identity in amino acid sequence) in 851 reported *A. baumannii* strains and with almost negligible homology to human proteins. Again, a recombinant Trx–Omp22 fusion protein showed 100% protection of immunised mice for seven days after lethal challenge at high doses, with lower doses showing protection of a third of the mice [159]. Although Omp22 has immunogenic and prophylactic properties, it may not be sufficiently potent to invoke adequate protection unless being administered at a high dose. Fusion of Omp22 with another weak antigen, OmpK, improved the protection against *A. baumannii* to a higher level than those immunised with either of the two proteins individually [160,161] demonstrating the utility of a multivalent vaccine approach.

#### 6.2.3. LOS and Conjugate Vaccines

A rather controversial and neglected antigen for subunit vaccine candidates is LOS (Section 5.2). OMPs extracted from *A. baumannii* cells or recombinant *A. baumannii* proteins produced by *E. coli* customarily require a detoxification step reducing the content of LOS to a minimal level to weaken the endotoxin activity, ensuring vaccine safety, and critically ensuring that the immunological response is mediated by the protein antigen being evaluated. LOS is also not a popular target for vaccine development due to the short-lasting B cell memory it induces; however, mice vaccinated with LOS alone were protected from a lethal challenge [142]. LOS is recognised by TLRs, particularly TLR-4, and safer derivatives may be feasible adjuvants. This has been supported by a study where LOS-free OMPs developed from an *A. baumannii* mutant deficient in LOS production showed significantly reduced protection (40%) compared with those derived from the wild-type cells, and administration of exogenous LOS restored the survival levels [142]. Noticeably, García-Quintanilla et al. reported that *A. baumannii* can acquire resistance to colistin and collateral sensitivity to azithromycin, rifampicin, and vancomycin through the complete loss of LOS, suggesting that LOS-based vaccines may deliver weakened protection against some problematic LOS-deficient *A. baumannii* MDR or PDR strains [142]. Despite this, although it may not be applicable as an immunogen, LOS (or safer derivatives) represent potentially powerful adjuvants boosting protective immune responses, and its usage as well as potential risks should be evaluated more comprehensively.

#### 6.2.4. Multi-Component Vaccines

Unfortunately, despite encouraging results in pre-clinical studies, single subunit antigens are unlikely to elicit comprehensive protection in humans against heterologous *A. baumannii* strains. To date, single recombinant protein vaccines have performed poorly in providing protection against bacteria that mediate pathogenesis via multiple virulence mechanisms. *A. baumannii* expresses several virulence factors that allow successful evasion of innate immune defence [168], contributing to the challenge of selecting appropriate antigens. Vaccines containing multiple antigens will display a higher density of epitopes with enriched diversity and are expected to exhibit increased immunogenicity, bringing better protection efficiency. Multi-component vaccines against *A. baumannii* are insufficiently studied and most of them are outer member complex (OMC) or outer membrane vesicle (OMV) based.

OMPs extracted from *A. baumannii* reference strain ATCC 19606 effectively elicited both humoral and cellular responses in vaccinated mice and induced 80% to 100% protection against sepsis following challenge with heterologous *A. baumannii* isolates. Notably, the antisera elicited with this OMC vaccine also cleared established *A. baumannii* infections (70–100% recovery rate) [136]. However, a drawback of these preparations is that the composition can be ill-defined, leading to major concerns on safety, regulatory compliance, and feasibility for consistent industrial production.

Despite this, the use of naturally secreted outer membrane vesicles (OMV) as vaccine technology platforms has gained renewed interest in recent years particularly since the approval of meningococcal group B vaccine, Bexsero^®^, an OMV-based product [169]. Crucially they present a wider variety of antigens, including both OMPs and other membrane-associated proteins, and can be further designed to package and deliver antigens. Consequently, with optimal bioengineering design, isolation, LOS detoxification, OMVs could be developed into safe, effective, and cost-effective vaccines against bacterial infections. This topic is covered in two excellent reviews [169,170]. Natural OMVs produced by the *A. baumannii* reference strain ATCC 19606 proved highly effective in immunising mice, and 90% of the immunised mice were protected against heterologous strains. Subsequent MALDI–TOF MS analysis identified six proteins with high immunogenicity in the OMVs, including previously mentioned, OmpA, OmpW, Omp33–36, and CarO, as well as two other putative proteins [138] which were likely critical to the protective response. In a later study, Huang et al. successfully engineered *E. coli*-derived OMVs packaging fused proteins of exogenous *A. baumannii* Omp22 and a pore-forming haemolytic protein ClyA, which protected over 60% and 100% of mice against a sepsis challenge, without any additional adjuvants. It is worth noting that the carrier control groups administered unmodified *E. coli* OMVs showed 35–63.6% survival indicating that *E. coli*-derived OMVs elicited cross-immunity to *A. baumannii* infection [162].

#### 6.2.5. Nucleic Acid Vaccines

Despite nucleic acid vaccines have gained increasing interest as vaccine platforms in recent years, there are barely any studies on nucleic acid vaccines against *A. baumannii*. Very recently, DNA vaccines delivering *A. baumannii* OmpA gene were reported to induce a mixed humoral and cellular response protecting lethal bacterial challenges in murine pneumonia models [163,164]. Recently, a pVAX1 vector encoding *A. baumannii* OmpA and proteoglycan associated lipoprotein (PAL) genes administered intramuscularly resulted in the protection of >80% of immunised mice to lethal pulmonary challenge with four heterologous strains, while immunisation with an OmpA encoding vector elicited 50% survival in response to challenge. A high level of humoral responses and mixed Th1/Th2/Th17 cellular responses, and reduced bacterial loads were reported with reduced inflammatory cytokines and inflammatory cell infiltration in the BAL [163]. Immunisation with an alternative plasmid-encoded-*ompA* vaccine utilising a eukaryotic expression vector pBudCE4.1 protected 60% of the immunised mice against pulmonary infection for up to 15 days post-challenge, eliciting moderate Il-2, IL-4, Il-12, and IFN-γ responses [164]. These nucleic vaccine approaches are still at an early stage but hold promise as safe cost-effective methods of administering multivalent vaccines.

### 6.3. Lessons from A. baumannii Vaccines and Future Directions

Despite the aforementioned efforts, the progress of vaccines against *A. baumannii* has lagged behind that for nosocomial pathogens such as *C. difficile*, *P. aeruginosa,* and *S. aureus* [9], and to date no candidate has entered clinical trials, suggesting the challenges in developing safe and efficacious *A. baumannii* vaccines.

#### 6.3.1. Modern Vaccine Technologies

Current investigations on anti-*A. baumannii* vaccines are still in their infancy, with most research efforts looking into conventional approaches and platforms, e.g., subunit and killed vaccines. Novel antigen carriers and advanced delivery systems, such as nucleic acid vaccines, virus vectors, conjugated carriers, and co-delivery of multiple antigens are rarely explored. Nevertheless, as the mRNA and DNA vaccine technologies have been acknowledged throughout the world during the COVID-19 vaccine race, it is foreseeable that burgeoning attention may be paid to nucleic acid vaccines against *A. baumannii* and other pathogens in the coming future. In addition, proteomic approaches and reverse vaccinology exploit modern bioinformatics, allowing the systematic in silico evaluation and selection of putative immunogens for vaccine candidates, which may have a wider application for future antigen selection and vaccine design against *A. baumannii* infections.

#### 6.3.2. Long-Term Cellular Immunity

In pre-clinical trials, a promising vaccine candidate against *A. baumannii* should demonstrate its protective benefits in immunised animals with a significantly improved survival rate, reduced bacterial burdens within organs, and the suppressed accumulation of inflammatory cytokines and chemokines in sera. Furthermore, the resulting anti-sera are expected to contain sufficient antibody titre for effective passive immunisation and even for antibody therapy treating established infections. Therefore, most studies have laid emphasis on evaluating post-immunisation or post-infection humoral immune responses, while cell-mediated immunity remains largely unstudied. However, as an invasive pathogen, it is essential that *A. baumannii* vaccines elicit dominant cellular immune responses and prime sustained T memory cells for long-term protection. In this context, though most studies reported robust vaccine-induced humoral or Th2-mediated immune responses, their performance in stimulating cellular immunity was not specified. Besides, the intervals between the last immunisation and bacterial challenge were only one week in some studies, making the evaluation of the endurance of the protective response even less reliable. It is hence important for the *A. baumannii* vaccine research community to standardise the evaluation protocols, such as the animal models, intervals between immunisation and challenges, challenge strains, doses, and routes, so that the comparison in the efficacy of the experimental vaccines between different laboratories is more rigorous [9].

#### 6.3.3. Multivalent Protection

It is encouraging to note that many *A. baumannii* vaccine studies have evaluated the protection against not only homologous strain challenges but also challenges raised by other clinical isolates to demonstrate the broad protection offered by the vaccine candidates. However, as the protection rates reported in these studies varied considerably with strain, immunisation, and/or challenging dose, their protective effects require more comprehensive evaluations covering a broader range of heterologous strains in diverse challenging scenarios, especially for those involving highly virulent strains and massive exposure dose. Strain-dependent diversity in the selected antigens may also increase the challenges in developing a vaccine providing broad or multivalent protection, as even in a specific bacterial strain, the amino acid sequences of some antigen proteins, e.g., OmpA, may vary among multiple subclasses [145,171]. At a high level, *A. baumannii* has an exceptional ability to acquire foreign DNA and generate new variants as the whole genome sequencing of ATCC 179878 found a significant fraction of open reading frames (ORF) (17.2%) in 28 putative alien islands [12], and such multiplicity of strains makes repeated infections possible and complicates vaccine development. Therefore, choosing conserved antigens and developing multi-component vaccines will be critical to effective protection.

## 7. Conclusions

The increasing attention to *A. baumannii* arose following outbreaks in the US army during the Iraq conflicts, and since then is becoming a global concern. Currently available treatment options for MDR *A. baumannii*-caused infections are quite limited where colistin is often reported as the only antibiotic delivering therapeutic effects, making the pathogen extremely difficult to treat. Massive research efforts have been devoted to its antibiotic resistance mechanisms and epidemiology but so far, our understanding of its pathology is comparably limited. In this context, vaccines against *A. baumannii* infections, are urgently needed and will be of great value in the prevention of infection and control of this canny pathogen. The past decade has witnessed the very first efforts in *A. baumannii* vaccine development, however, none have entered clinical trials, which is clear evidence of the challenges involved and the need for further progress in understanding the complex *A. baumannii*–host relationships at each stage of the infection process. Most *A. baumannii* vaccine candidates developed to date explored conventional protein-based technologies, inducing opsonophagocytic antibody-mediated killing and/or antibody-mediated toxin inhibition. However, a polarised Th2 response may not be sufficient for effective and long-term protection, considering that *A. baumannii* is an invasive pathogen; consequently, a mixed Th1/Th2 orTh1/Th17 response may be more beneficial. Investigations of novel platforms, as well as adjuvant development, could offer the possibility of optimising immune mechanisms and targeting multiple antigens, which will eventually deliver vaccines with broad coverage and clinical efficacy. In addition, the COVID vaccine and associated technology acceleration could change the landscape of future vaccine research and development. With the licensing of the first mRNA vaccines, nucleic acid vaccines against *A. baumannii* and other invasive pathogens are expected to generate more interest in the near future. There is some urgency, however, in order to tackle the global spread of MDR *A. baumannii*, and vaccine development needs to accelerate in order to keep up with the pace of spread.

## Figures and Tables

**Table 2:** Target Populations of *A. baumannii* Vaccines.

Susceptible Group ^1^	Examples	Vaccination Indication
Inpatients with immune deficiencies or compromising health conditions.	People with hematologic disorders, neutropenia, immune regulatory abnormalities, or receiving splenectomy, organ transplantation; patients kept in ICU or long-term hospitalisation, particularly those intubated.	Ideally vaccination before or soon after being admitted to hospital, especially in medical institutions with historical nosocomial outbreaks.
People who are more likely to suffer trauma or receive untimely, inappropriate wound treatments.	Soldiers or peacekeepers serving in ‘hot’ conflict regions; survivors from natural disasters and war; residents of temporary refugee settlements; employees with high-risk jobs such as logging, mining, fishing, emergency response.	Troops assigned to regions with high prevalence of *A. baumannii* should be vaccinated before deployment; large-scale vaccination in advance, unlikely, thus injured people with severe open wounds should be vaccinated as soon as possible after receiving first aid treatment.
People exposed to a high risk of community transmission.	Vulnerable people, e.g., the elderly, in communities with high skin-colonisation or environmental isolation rate, e.g., tropical areas in Asia–Pacific.	People may require vaccination more voluntarily.

^1^ Especially for areas of high prevalence and incidence.

## Data Availability

The data presented in this study is contained within the article and supplementary materials.

## Supplementary Materials

The following are available online at https://www.mdpi.com/article/10.3390/vaccines9060570/s1, Table S1: Type strains for the phylogenetic analysis of Acb complex, Table S2: Worldwide data for percentage of carbapenem-resistant *A. baumannii* (R%), Table S3: European data (2018) for R% and F%.
