# Supplementary material for: Mapping Global Prevalence of Acinetobacter baumannii and Recent Vaccine Development to Tackle It
Source: Vaccines (Basel). 2021 Jun 1;9(6):570. doi: 10.3390/vaccines9060570 (PMC8226933; doi:10.3390/vaccines9060570)
Supplement: Supplementary file 1 [file vaccines-09-00570-s001.zip › vaccines-1210259-supplementary.pdf]

Mapping Global Prevalence of the Opportunist *Acinetobacter baumannii* and Recent Vaccine Development to Tackle It

Supplementary Materials

Chaoying Ma and Siobhán McClean*

School of Biomolecular and Biomedical Sciences, University College Dublin, Belfield, Dublin 4, D04 V1W8, Ireland; chaoying.ma@ucdconnect.ie (C.M.)

* Correspondence: siobhan.mcclean@ucd.ie

Table S1. Type strains for the phylogenetic analysis of Acb complex

Species	Type Strain	GenBank Accession
A. baumannii	ATCC 19606	HE651907
A. calcoaceticus	ATCC 23055	AJ888984
A. nosocomialis	RUH 2376	NR_117931
A. pittii	DSM 21653	NR_116774
A. seifertii	LUH 1472	NR_134684
A. lactucae	NRRL B-41902	NR_152004
A. oleivorans	DR1	NR_102814
M. osloensis	DSM 6998	AB643599

Table S2. Worldwide data for percentage of carbapenem-resistant *A. baumannii* (R%)

**Country (Region)**	**Area**	**Sample Collected Year (2009-2019)**	**R%**	**Reference**
Armenia	Middle East	2018 Full Year	0%†	RA [1]
Belize	Latin America	2009-2017	0%	RA[1] [2]
Finland	Europe	2018 Full Year	0%*	SP[2] [3]
Iceland	Europe	2018 Full Year	0%†	SP [3]
Norway	Europe	2018 Full Year	0%	SP [3]
Togo	Africa	2011.01-2012.12	0%†	RA [4]
Ireland	Europe	2018 Full Year	1.8%	SP [3]
United Kingdom	Middle East	2018 Full Year	1.8%	SP [3]
Australia	Oceania	2017 Full Year	2.8%	SP [5]
Switzerland	Europe	2018 Full Year	3%	SP [1]
Japan	Asia	2018 Full Year	3.5%	SP [6]
Sweden	Europe	2018 Full Year	3.7%	SP [3]
Belgium	Europe	2018 Full Year	3.8%	SP [3]
Austria	Europe	2018 Full Year	4.4%	SP [3]
Germany	Europe	2018 Full Year	4.4%	SP [3]
Netherlands	Europe	2018 Full Year	4.6%	SP [3]
Canada	North America	2018 Full Year	4.7%	SP [7]
Denmark	Europe	2018 Full Year	6.4%	SP [3]
France	Europe	2018 Full Year	6.5%	SP [3]
New Zealand	Oceania	2016 Full Year	6.5%	SP [8]
Guyana	Latin America	2008.01-2014.12	7.5%	RA [9]
Sierra Leone	Africa	2018.02-2018.06	8.7%*	RA [10]
Cambodia	Asia	2007-2016	11.1%*	RA [11]
Namibia	Africa	2009.01-2012.05	12.5%†	RA [12]
Zimbabwe	Europe	2012.01-2017.12	15.4%	RA [13]
Benin	Africa	Specific Period Unknown	16%†	RA [14]
Equatorial Guinea	Africa	2013.07-2016.01	17.6%*	RA [15]
Slovenia	Europe	2018 Full Year	17.9%*	SP [3]
Czech Republic	Europe	2018 Full Year	19.8%	SP [3]
Dominican Republic	Latin America	2013 Full Year	20%	SP [16]
Cameroon	Africa	2005-2014	21.1%	RA [17]
Honduras	Latin America	2013 Full Year	23%	SP [16]
Grenada	Latin America	2015-2017	25%	RA [18]
Jamaica	Latin America	2009.05-2010.04	25.6%	RA [19]
Estonia	Europe	2018 Full Year	28.6%*	SP [3]
United Arab Emirates	Middle East	2017 Full Year	29.4%	SP [20]
Portugal	Europe	2018 Full Year	30.7%	SP [3]
Ethiopia	Africa	2018.04-2018.07	33.3%†	RA [21]
El Salvador	Latin America	2013	38%	SP [16]
Uganda	Africa	2007.02-2009.09	38%	RA [22]
Singapore	Asia	2017 Full Year	43.2%	SP [23]
Bangladesh	Asia	2017.01-2018.01	43.5%	RA [24]
Madagascar	Africa	2006.09-2009.03	44%	RA [25]

Slovakia	Europe	2018 Full Year	44.0%	SP [3]
Qatar	Middle East	2012.01-2013.12	45.6%	RA [26]
Algeria	Africa	2011.03-2011.07	47.9%	RA [27]
United States	North America	2009-2012	47.9%	RA [28]
Chile	Latin America	2010 Full Year	48%	SP [29]
Spain	Europe	2018 Full Year	54.3%	SP [3]
Hungary	Europe	2018 Full Year	55.2%	SP [3]
Philippines	Asia	2018 Full Year	56%	SP [30]
Bolivia	Latin America	2013 Full Year	57%	SP [16]
Guatemala	Latin America	2013 Full Year	57%	SP [16]
Malaysia	Asia	2017 Full Year	61.2%	SP [31]
Uruguay	Latin America	2010 Full Year	64%	SP [29]
Pakistan	Middle East	2013-2015	65%	SP [32]
Poland	Europe	2018 Full Year	67.3%	SP [3]
Thailand	Asia	2018 Full Year	68.2%	SP [33]
Egypt	Africa	Fall and Winter 2010 Spring 2015	70.3%	RA [34]
Syria	Middle East	2008.01-2011.06	70.5%	RA [35]
Taiwan	Asia	2018 Full Year	71.3%	SP [36]
Kenya	Africa	2013-2015	72.8%	RA [37]
Venezuela	Latin America	2013 Full Year	71%	SP [16]
Mauritius	Africa	2014.07	74%	RA [38]
Saudi Arabia	Middle East	2017 Full Year	74%	SP [39]
Bulgaria	Europe	2018 Full Year	74.5%	SP [3]
Ukraine	Europe	2018 Full Year	75%*	SP [1]
China	Asia	2019 Full Year	75.1%	SP [40]
Morocco	Africa	2012.04-2014.04	76%	RA [41]
Lebanon	Middle East	2013.10-2015.12	76.5%	RA [42]
Paraguay	Latin America	2013 Full Year	77%	SP [16]
Brazil	Latin America	2017 Full Year	77.7%	SP [43]
Israel	Middle East	2017 Full Year	78%	RA [44]
North Macedonia	Europe	2018 Full Year	78%*	SP [1]
Panama	Latin America	2013 Full Year	78%	SP [16]
Latvia	Europe	2018 Full Year	78.4%	SP [3]
Moldova	Europe	Specific Period Unknown	78.7%	RA [45]
Albania	Europe	2018.02-2018.05	78.9%*	RA [46]
Nicaragua	Latin America	2013 Full Year	80%	SP [16]
Russia	Europe	2018 Full Year	79%	SP [1]
Italy	Europe	2018 Full Year	79.2%	SP [3]
Mexico	North America	2018.01-2018.06	79.6%	RA [47]
Indonesia	Asia	2017 Full Year	80.0%	SP [48]
South Africa	Africa	2017 Full Year	81%	SP [49]
Iran	Middle East	2001-2013	81.5%	RA [50]
Argentina	Latin America	2018 Full Year	82.9%	SP [51]
Ecuador	Latin America	2013 Full Year	83%	SP [16]
Cuba	Latin America	2013 Full Year	84%	SP [16]

Iraq	Middle East	2014.12-2015.05	84%	RA [52]
Cyprus	Europe	2018 Full Year	84.2%	SP [3]
Vietnam	Asia	2012-2014	84.4%	RA [53]
Kazakhstan	Asia	2012-2015	84.5%	RA [54]
India	Asia	2019 Full Year	84.8%	SP [55]
Oman	Middle East	2018 Full Year	84.9%	SP [56]
Romania	Europe	2018 Full Year	85.3%	SP [3]
Nigeria	Africa	Specifical Period Unknown	85.7%*	RA [57]
Montenegro	Europe	2018 Full Year	86%*	SP [1]
Nepal	Asia	2017 Full Year	86%	RA [58]
Kuwait	Middle East	2015.03-2016.06	87.0%	RA [59]
Tunisia	Africa	2013-2016	87.1%	RA [60]
Libya	Africa	2013.04-2014.04	87.8%*	RA [61]
Rwanda	Africa	2013 Full Year	88%	RA [62]
Georgia	Europe	2018 Full Year	89%	SP [1]
Kosovo	Europe	2018 Full Year	89%	SP [1]
Sudan	Africa	2011.07-2014.08	89%	RA [63]
Lithuania	Europe	2018 Full Year	89.8%	SP [3]
Yemen	Middle East	2012.10-2013.09	90%	RA [64]
Turkey	Europe	2018 Full Year	92%	SP [1]
South Korea	Asia	2017 Full Year	92.1%	SP [65]
Greece	Europe	2018 Full Year	92.4%	SP [3]
Bosnia and Herzegovina	Europe	2018 Full Year	93%	SP [1]
Belarus	Europe	2018 Full Year	94%	SP [1]
Croatia	Europe	2018 Full Year	95.5%	SP [3]
Serbia	Europe	2018 Full Year	96%	SP [1]
Colombia	Latin America	2019 Full Year	97.0%	SP [66]
Jordan	Middle East	2009.05-2010.02	97%	RA [67]
Peru	Latin America	2014.02-2016.04	97.5%	RA [68]
Palestine	Middle East	2006.01-2014.02	98%	RA [69]
Botswana	Africa	2017 Full Year	100%*	RA [70]
Ghana	Africa	2015.02-2015.08	100%†	RA [71]
Myanmar	Asia	2015.12-2018.01	100%	RA [72]
Sri Lanka	Asia	2013 Full Year	100%*	SP [73]
Timor-Leste	Asia	2017.03-2017.06	100%†	RA [74]
Armenia	Middle East	N/A[3]		
Azerbaijan	Middle East	N/A		
Brunei Darussalam	Asia	N/A		
Laos	Asia	N/A		
Luxembourg	Europe	N/A		
Malta	Europe	N/A		
Afghanistan	Middle East	N/A		
Andorra	Europe	N/A		
Angola	Africa	N/A		
Antigua and Barbuda	Latin America	N/A		

Bahamas	Latin America	N/A
Bahrain	Middle East	N/A
Barbados	Latin America	N/A
Bhutan	Asia	N/A
Burkina Faso	Africa	N/A
Burundi	Africa	N/A
Cabo Verde	Africa	N/A
Central African Republic	Africa	N/A
Chad	Africa	N/A
Comoros	Africa	N/A
Congo (Congo-Brazzaville)	Africa	N/A
Costa Rica	Latin America	N/A
Cote d'lvoire	Africa	N/A
Democratic Republic of the Congo	Africa	N/A
Djibouti	Africa	N/A
Dominica	Latin America	N/A
Eritrea	Africa	N/A
Eswatini (Swaziland)	Africa	N/A
Fiji	Oceania	N/A
Gabon	Africa	N/A
Gambia	Africa	N/A
Guinea	Africa	N/A
Haiti	Latin America	N/A
Kiribati	Oceania	N/A
Kyrgyzstan	Asia	N/A
Lesotho	Africa	N/A
Liberia	Africa	N/A
Liechtenstein	Europe	N/A
Malawi	Africa	N/A
Maldives	Asia	N/A
Mali	Africa	N/A
Marshall Islands	Oceania	N/A
Mauritania	Africa	N/A
Micronesia	Oceania	N/A
Monaco	Europe	N/A
Mongolia	Asia	N/A
Mozambique	Africa	N/A
Nauru	Oceania	N/A
Niger	Africa	N/A
North Korea	Asia	N/A
Palau	Oceania	N/A
Papua New Guinea	Oceania	N/A
Saint Kitts and Nevis	Latin America	N/A

Saint Lucia	Latin America	N/A
Saint Vincent and the Grenadines	Latin America	N/A
Samoa	Oceania	N/A
San Marino	Europe	N/A
Sao Tome and Principe	Africa	N/A
Senegal	Africa	N/A
Seychelles	Africa	N/A
Solomon Islands	Oceania	N/A
Somalia	Africa	N/A
South Sudan	Africa	N/A
Suriname	Latin America	N/A
Tajikistan	Asia	N/A
Tanzania	Africa	N/A
Tonga	Oceania	N/A
Trinidad and Tobago	Latin America	N/A
Turkmenistan	Asia	N/A
Tuvalu	Oceania	N/A
Uzbekistan	Asia	N/A
Vanuatu	Oceania	N/A
Vatican	Europe	N/A
Zambia	Africa	N/A

1. Data sources are national or regional AMR surveillance programs.

2. Data sources are research or review articles.

3. No surveillance or research data available for the selected time range.

* A small number of isolates was tested (n < 30), and the percentage resistance should be interpreted with caution.

† Less than 10 isolates reported, and the percentage resistance should be interpreted with extreme caution.

R% = (Number of Carbapenem-resistant *A. baumannii* Isolates) / (Number of *A. baumannii* Isolates)

The sample collecting methods, susceptibility testing methods and evaluating criteria may vary in different countries (regions). Antibiotics used in the susceptibility tests usually are imipenem (10 μg for disk diffusion test) and meropenem (10 μg).

Table S3. European data (2018) for R% and F%

Country (Region)	R%	F% Data sources: [1, 3]
Armenia	0%	2.27%
Finland	0.0%	0.28%
Iceland	0.0%	0.52%
Norway	0.0%	0.41%
Togo	0%	3.00%
Ireland	1.7%	0.96%
United Kingdom	1.8%	1.21%
Switzerland	3%	0.60%
Sweden	3.7%	0.44%
Belgium	3.8%	1.26%
Austria	4.4%	0.73%
Germany	4.4%	1.10%
Netherlands	4.6%	0.86%
Denmark	6.4%	0.48%
France	6.5%	1.69%
Slovenia	17.9%	1.17%
Czech Republic	19.8%	0.97%
Estonia	28.6%	0.79%
Portugal	30.7%	0.78%
Slovakia	44.0%	4.88%
Spain	54.3%	0.49%
Hungary	55.2%	4.86%
Poland	67.3%	3.62%
Bulgaria	74.5%	8.59%
Ukraine	75%	18.71%
North Macedonia	78%	10.23%
Latvia	78.4%	4.16%
Russia	79%	15.11%
Italy	79.2%	3.23%
Cyprus	84.2%	9.72%
Romania	85.3%	8.91%
Montenegro	86%	10.29%
Georgia	89%	15.41%
Kosovo	89%	34.50%
Lithuania	89.8%	3.27%
Turkey	92%	13.33%
Greece	92.4%	14.26%
Bosnia and Herzegovina	93%	12.91%
Belarus	94%	21.97%
Croatia	95.5%	5.69%
Serbia	96%	18.67%

Reference

1. WHO Regional Office for Europe, Central Asian and European Surveillance of Antimicrobial Resistance Annual Report 2019. 2019.
2. Tuyud, V. and T. Thiagarajan, Antimicrobial Susceptibility Patterns of Pathogens Isolated from Surgical Site Infections at Public Health Facilities in Belize. BIOLOGY THESES REPOSITORY, 2019. 1(1).
3. European Centre for Disease Prevention and Control, Surveillance of antimicrobial resistance in Europe 2018: Annual report of the European Antimicrobial Resistance Surveillance Network (EARS-Net). 2019.
4. Bossa, Y., et al., Antibiotic resistance profile of bacteria isolated from patients admitted with postoperative infection in a regional hospital center in Togo. African Journal of Microbiology Research, **2014**. 8(44): p. 3702-3709.
5. Australian Commission on Safety and Quality in Health Care, Third Australian report on antimicrobial use and resistance in human health. 2019.
6. 薬剤耐性ワンヘルス動向調査検討会, 薬剤耐性ワンヘルス動向調査年次報告書 2019 Nippon AMR One Health Report (NAOR) 2019. 2019, 日本厚生労働省.
7. Public Health Agency of Canada, Canadian Antimicrobial Resistance Surveillance System Report. 2020, Public Health Agency of Canada.
8. Science for Communities, New Zealand Public Health Surveillance Report: Antimicrobial susceptibility data from hospital and community laboratories, 2017. 2017, New Zealand Ministry of Health.
9. Mahamat, A., et al., Clinical epidemiology and resistance mechanisms of carbapenem-resistant Acinetobacter baumannii, French Guiana, 2008-2014. Int J Antimicrob Agents, **2016**. 48(1): p. 51-55.
10. Lakoh, S., et al., Antibiotic resistance in patients with clinical features of healthcare-associated infections in an urban tertiary hospital in Sierra Leone: a cross-sectional study. Antimicrob Resist Infect Control, **2020**. 9(1): p. 38.
11. Fox-Lewis, A., et al., Antimicrobial Resistance in Invasive Bacterial Infections in Hospitalized Children, Cambodia, 2007-2016. Emerg Infect Dis, **2018**. 24(5): p. 841-851.
12. Mengistu, A., et al., Antimicrobial sensitivity patterns of cerebrospinal fluid (CSF) isolates in Namibia: implications for empirical antibiotic treatment of meningitis. Journal of pharmaceutical policy and practice, **2013**. 6(1): p. 4.
13. Mhondoro, M., et al., Trends in antimicrobial resistance of bacterial pathogens in Harare, Zimbabwe, 2012–2017: a secondary dataset analysis. BMC Infectious Diseases, **2019**. 19(1): p. 1-9.
14. Dougnon, V.T., et al., Infection Risks and Antimicrobial Resistance in Tertiary Hospitals in Benin: Study Cases of Sakété-Ifangni and Menontin Hospitals. International Journal of Infection, **2020**. 7(1).
15. Shatalov A, et al., Low Efficiency of the Commonly Prescribed Drugs against Klebsiella pneumonia, Escherichia coli and Acinetobacter Species as the Causative Agents of Blood Stream Infection in Malabo, Equatorial Guinea. Advances in Microbiology. **2016**;6(03):162.
16. Sociedade Brasileira de Parasitologia, Revista de Patologia Tropical: Red Latinoamericana de Vigilancia de la Resistencia a los Antimicrobianos. 2014, Instituto de Patologia Tropical Sociedade Brasileira de Parasitologia.
17. Okalla Ebongue, C., Antimicrobial Multi-Resistance of Acinetobacter baumanii Isolated from Clinical Specimens in Douala (Cameroon). Journal of Diseases and Medicinal Plants, **2015**. 1(2): p. 31.
18. Sharma, D., S.E. Preston, and R. Hage, Emerging Antibiotic Resistance to Bacterial Isolates from Human Urinary Tract Infections in Grenada. Cureus, **2019**. 11(9): p. e5752.
19. Thoms-Rodriguez, C., et al., Detection of OXA Carbapenemase Positive Acinetobacter spp., Jamaica. West Ind Med J, **2016**.
20. UAE AMR Surveillance Sub-Committee, Updates on AMR Surveillance in the UAE. in UAE International Conference on Antimicrobial Resistance. 2018.
21. Motbainor, H., F. Bereded, and W. Mulu, Multi-drug resistance of blood stream, urinary tract and surgical site nosocomial infections of Acinetobacter baumannii and Pseudomonas aeruginosa among patients hospitalized at Felegehiwot referral hospital, Northwest Ethiopia: a cross-sectional study. BMC Infect Dis, **2020**. 20(1): p. 92.
22. Kateete, D.P., et al., Carbapenem resistant Pseudomonas aeruginosa and Acinetobacter baumannii at Mulago Hospital in Kampala, Uganda (2007–2009). Springerplus, 2016. 5(1): p. 1308.
23. One Health Antimicrobial Resistance Working Group, One Health Report on Antimicrobial Utilisation and Resistance, 2017. 2019, National Centre for Infectious Diseases.
24. Ifa, I., et al., Isolation of Acinetobacter species from Clinical Specimens with Detection of Their Antimicrobial Susceptibility Pattern from a Tertiary Care Hospital, Bangladesh. Mymensingh Medical Journal: MMJ, **2020**. 29(3): p. 622-627.
25. Andriamanantena, T.S., et al., Dissemination of multidrug resistant Acinetobacter baumannii in various hospitals of Antananarivo Madagascar. Annals of clinical microbiology and antimicrobials, **2010**. 9(1): p. 17.
26. Al Samawi, M.S., et al., Acinetobacter Infections among Adult Patients in Qatar: A 2-Year Hospital-Based Study. Can J Infect Dis Med Microbiol, **2016**. 2016: p. 6873689.
27. Bakour, S., et al., Antibiotic resistance determinants of multidrug-resistant Acinetobacter baumannii clinical isolates in Algeria. Diagnostic Microbiology and Infectious Disease, **2013**. 76(4): p. 529-531.
28. Zilberberg, M.D., M.H. Kollef, and A.F. Shorr, Secular trends in Acinetobacter baumannii resistance in respiratory and blood stream specimens in the United States, 2003 to 2012: A survey study. J Hosp Med, **2016**. 11(1): p. 21-6.
29. Sociedade Brasileira de Parasitologia, Revista de Patologia Tropical: Red Latinoamericana de Vigilancia de la Resistencia a los Antimicrobianos. 2011, Instituto de Patologia Tropical Sociedade Brasileira de Parasitologia.

30. ARSP, Antimicrobial Resistance Surveillance Program 2018 Annual Report. 2019, Department of Health (Philippines), Reserach Insititute for Tropical Medicine, Antimicrobial Resistance Surveillance Research Laboratory.
31. Antibiotic Resistance Surveillance Reference Laboratory, National Antibiotic Resistance Surveillance Annual Report 2017. 2018, Bacteriology Unit, Infectious Diseases Research Centre, Institute for Medical Research.
32. Global Antibiotic Resistance Partnership, Situation Analysis Report on Antimicrobial Resitance in Pakistan: Findings and Recommendations for Antibiotics Use and Resistance. 2017, Center for Disease Dynamics, Econmoics & Policy.
33. Styles, K.M., et al., Investigating Bacteriophages Targeting the Opportunistic Pathogen Acinetobacter baumannii. Antibiotics (Basel), **2020**. 9(4).
34. Abouelfetouh, A., A.S. Torky, and E. Aboulmagd, Phenotypic and genotypic characterization of carbapenem-resistant Acinetobacter baumannii isolates from Egypt. Antimicrob Resist Infect Control, **2019**. 8: p. 185.
35. Hamzeh, A.R., M. Al Najjar, and M. Mahfoud, Prevalence of antibiotic resistance among Acinetobacter baumannii isolates from Aleppo, Syria. Am J Infect Control, **2012**. 40(8): p. 776-7.
36. 台灣院內感染監視資訊系統（TNIS），邁向全球衛生安全-抗生素抗藥性管理行動策略計畫 (109 年至 113 年）. 2019, 衛生福利部疾病管制署.
37. Musyoki, V.M., et al., Antimicrobial susceptibility pattern of Acinetobacter isolates from patients in Kenyatta National Hospital, Nairobi, Kenya. The Pan African Medical Journal, **2019**. 33.
38. Issack, M., Antibiotic resistance among hospitalized patients in Mauritius in 2014. International Journal of Infectious Diseases, **2016**. 45: p. 94.
39. Health, S.A.M.o., Summary Report of Antibiogram Data from MOH Hospitals (2017). 2018.
40. CHINET 中国细菌耐药监测网, 2019 年 CHINET 三级医院细菌耐药监测. Available online: http://www.chinets.com/Data/AntibioticDrugFast (accessed on 14 September 2020).
41. Uwingabiye, J., et al., Acinetobacter infections prevalence and frequency of the antibiotics resistance: comparative study of intensive care units versus other hospital units. Pan Afr Med J, **2016**. 23: p. 191.
42. Al Atrouni, A., et al., Wide spread of OXA-23-producing carbapenem-resistant Acinetobacter baumannii belonging to clonal complex II in different hospitals in Lebanon. Int J Infect Dis, **2016**. 52: p. 29-36.
43. Agência Nacional de Vigilância Sanitária, Boletim Segurança do Paciente e Qualidade em Serviços de Saúde Nº 17: Incidentes Relacionados à Assistência à Saúde 2017. 2018, Brazilian Health Regulatory Agency.
44. Dickstein, Y., et al., Trends in antimicrobial resistance in Israel, 2014-2017. Antimicrob Resist Infect Control, **2019**. 8: p. 96.
45. Prisacari, V., et al., Rezultatele studiului de evaluare a antibioticorezistenței microbiene în condițiile Republicii Moldova. 2016.
46. Namiganda, V., et al., Antibiotic Resistance Pattern of Acinetobacter baumannii Strains Isolated from Different Clinical Specimens and Their Sensibility Against Bioactive Molecules Produced by Actinobacteria. Arabian Journal for Science and Engineering, **2019**. 44(7): p. 6267-6275.
47. Garza-Gonzalez, E., et al., A snapshot of antimicrobial resistance in Mexico. Results from 47 centers from 20 states during a six-month period. PLoS One, **2019**. 14(3): p. e0209865.
48. Dahesihdewi, A., A.K. Sugianli, and I. Parwati, The surveillance of antibiotics resistance in Indonesia: a current reports. Bali Medical Journal, **2019**. 8(2): p. 565.
49. National Department of Health, Surveillance for Antimicrobial Resistance and Consumption of Antibiotics in South Africa. 2018.
50. Moradi, J., F.B. Hashemi, and A. Bahador, Antibiotic Resistance of Acinetobacter baumannii in Iran: A Systemic Review of the Published Literature. Osong Public Health Res Perspect, **2015**. 6(2): p. 79-86.
51. Argentine Ministry of Health, Vigilancia de la Resistencia a los Antimicrobianos: Red Latinoamericana de Vigilancia de la Resistencia a los Antimicrobianos. 2018.
52. Al-Samaree, M.Y. and Z.M. Al-Khafaji, Antibiogram of Acinetobacter baumannii isolated from Baghdad Hospitals. Int. J. Adv. Res. Biol. Sci, **2016**. 3(4): p. 238-242.
53. Anh, N.T., et al., Molecular epidemiology and antimicrobial resistance phenotypes of Acinetobacter baumannii isolated from patients in three hospitals in southern Vietnam. Journal of medical microbiology, **2017**. 66(1): p. 46-53.
54. Azizov, I., et al., The sensitivity to antibiotics of nosocomial strains of acinetobacter baumanii isolated in the tertiary hospitals in the Central Kazakhstan. International Journal of Infectious Diseases, **2016**. 45: p. 126.
55. Walia, K., V. Ohri, and A.K. Sahni, Annual Report Antimicrobial Resistance Surveillance and Research Network. 2020, Indian Council of Meidcal Research.
56. Directorate General for Diseases Surveillance and Control, Oman Antimicrobial Resistance Surveillance System Annual Report 2018. 2019, Ministry of Health, Oman.
57. Bashir, A., et al., Molecular characterization of Acinetobacter baumannii from patients with prolonged hospital stays in three tertiary hospitals of Kano Metropolis, Northwestern Nigeria. African Journal of Microbiology Research, **2019**. 13(27): p. 510-517.
58. Yadav, S.K., et al., Burden of Multidrug-Resistant Acinetobacter baumannii Infection in Hospitalized Patients in a Tertiary Care Hospital of Nepal. Infect Drug Resist, **2020**. 13: p. 725-732.
59. Al-Hashem, G., V.O. Rotimi, and M.J. Albert, Antimicrobial Resistance of Serial Isolates of Acinetobacter baumannii Colonizing the Rectum of Adult Intensive Care Unit Patients in a Teaching Hospital in Kuwait. Microbial Drug Resistance, 2020.

60. Cheikh, H.B., et al., Molecular characterization of carbapenemases of clinical Acinetobacter baumannii–calcoaceticus complex isolates from a University Hospital in Tunisia. 3 Biotech, **2018**. 8(7): p. 297.
61. Mathlouthi, N., et al., Emergence of carbapenem-resistant Pseudomonas aeruginosa and Acinetobacter baumannii clinical isolates collected from some Libyan hospitals. Microbial Drug Resistance, **2015**. 21(3): p. 335-341.
62. Carroll, M., et al., Five-Year Antimicrobial Susceptibility Trends Among Bacterial Isolates from a Tertiary Health-Care Facility in Kigali, Rwanda. Am J Trop Med Hyg, **2016**. 95(6): p. 1277-1283.
63. Omer, M.I., et al., Prevalence and resistance profile of Acinetobacter baumannii clinical isolates from a private hospital in Khartoum, Sudan. Am J Microbiol Res, **2015**. 3(2): p. 76-9.
64. Zabada, A.A.M., et al., Acinetobacter baumannii complex and its antibiotics susceptibility in selected hospital's intensive care units at Sana'a city-Yemen. Glob Adv Res J Microbiol, **2018**(3): p. 48-56.
65. Liu, C., et al., Antimicrobial resistance in South Korea: A report from the Korean global antimicrobial resistance surveillance system (Kor-GLASS) for 2017. Journal of Infection and Chemotherapy, **2019**. 25(11): p. 845-859.
66. Melendez R. and Jenifer C., Susceptibilidad antibiótica de bacterias aisladas de urocultivos de pacientes atendidos en el Hospital Regional Docente de Trujillo–La Libertad, 2019. **2019**.
67. Obeidat, N., et al., Major biologic characteristics of Acinetobacter baumannii isolates from hospital environmental and patients' respiratory tract sources. Am J Infect Control, **2014**. 42(4): p. 401-4.
68. Levy-Blitchtein, S., et al., Emergence and spread of carbapenem-resistant Acinetobacter baumannii international clones II and III in Lima, Peru. Emerg Microbes Infect, **2018**. 7(1): p. 119.
69. Handal, R., et al., Characterization of Carbapenem-Resistant Acinetobacter baumannii Strains Isolated from Hospitalized Patients in Palestine. Int J Microbiol, 2017. **2017**: p. 8012104.
70. Mpinda-Joseph, P., et al., Healthcare-associated infections including neonatal bloodstream infections in a leading tertiary hospital in Botswana. Hosp Pract (1995), **2019**. 47(4): p. 203-210.
71. Agyepong, N., et al., Multidrug-resistant gram-negative bacterial infections in a teaching hospital in Ghana. Antimicrob Resist Infect Control, **2018**. 7: p. 37.
72. Tada, T., et al., Molecular epidemiology of multidrug-resistant Acinetobacter baumannii isolates from hospitals in Myanmar. J Glob Antimicrob Resist, **2020**. 22: p. 122-125.
73. Sri Lanka College of Microbiologists, National Surveillance of Antimicrobial Resistance Report to Ministry of Health. 2014, SLCM ARSP & NLBSA Technical Committees.
74. Marr, I., et al., Antimicrobial resistance in urine and skin isolates in Timor-Leste. J Glob Antimicrob Resist, **2018**. 13: p. 135-138.
